# Glutamine Acts as a Neuroprotectant against DNA Damage, Beta-Amyloid and H_2_O_2_-Induced Stress

**DOI:** 10.1371/journal.pone.0033177

**Published:** 2012-03-08

**Authors:** Jianmin Chen, Karl Herrup

**Affiliations:** Department of Cell Biology and Neuroscience, Rutgers University, Piscataway, New Jersey, United States of America; Case Western Reserve University, United States of America

## Abstract

Glutamine is the most abundant free amino acid in the human blood stream and is ‘conditionally essential’ to cells. Its intracellular levels are regulated both by the uptake of extracellular glutamine via specific transport systems and by its intracellular synthesis by glutamine synthetase (GS). Adding to the regulatory complexity, when extracellular glutamine is reduced GS protein levels rise. Unfortunately, this excess GS can be maladaptive. GS overexpression is neurotoxic especially if the cells are in a low-glutamine medium. Similarly, in low glutamine, the levels of multiple stress response proteins are reduced rendering cells hypersensitive to H_2_O_2_, zinc salts and DNA damage. These altered responses may have particular relevance to neurodegenerative diseases of aging. GS activity and glutamine levels are lower in the Alzheimer's disease (AD) brain, and a fraction of AD hippocampal neurons have dramatically increased GS levels compared with control subjects. We validated the importance of these observations by showing that raising glutamine levels in the medium protects cultured neuronal cells against the amyloid peptide, Aβ. Further, a 10-day course of dietary glutamine supplementation reduced inflammation-induced neuronal cell cycle activation, tau phosphorylation and ATM-activation in two different mouse models of familial AD while raising the levels of two synaptic proteins, VAMP2 and synaptophysin. Together, our observations suggest that healthy neuronal cells require both intracellular and extracellular glutamine, and that the neuroprotective effects of glutamine supplementation may prove beneficial in the treatment of AD.

## Introduction

Glutamine is the most abundant free amino acid in the human blood stream. It is typically classified as a ‘non-essential’ amino acid because it can be made from TCA metabolites by most cells. A more accurate classification of the body's need for glutamine, however, would be the term ‘conditionally essential’. Many cell types are unable to survive in the complete absence of glutamine. Indeed, in certain B-cell lines supranormal concentrations are required. The value of glutamine is particularly apparent during stress. It becomes essential in organs or organ systems weakened by sickness, surgery or injury. Glutamine can regulate a variety of target genes involved in cell proliferation, differentiation and survival. It accomplishes this by altering the behavior of a range of transcription factors, including NFκB, through which the anti-inflammatory role of glutamine may be mediated [1]. A molecular explanation for the broad cellular dependence on glutamine remains elusive, but a key insight has emerged from recent studies showing that high intracellular glutamine is rate limiting for the uptake of several essential amino acids through the SLC7A5/SLC3A2 bidirectional transporter [2].

In brain, the majority of endogenous glutamine is produced by glutamine synthetase (GS), which catalyzes the formation of glutamine from glutamate and ammonia. Although all cells express GS to some extent, in the adult brain its levels are 40-fold higher in astrocytes than in neurons [3], [4], [5]. In adult brains, GS is neuroprotective [6], [7] and during embryogenesis functional GS is crucial for brain development. This can be seen from the finding that congenital GS deficiency causes brain malformation and neonatal death both in human and in mouse [8], [9]. GS responds to a variety of insults including oxidative stress, inflammation, and viral infection [10], [11], [12], [13], [14], suggesting a connection to neurodegenerative disease. Indeed, changes in GS level, activity and modifications have been documented in AD patients. Monomeric GS protein was found in 38 of 39 cerebrospinal fluid (CSF) samples obtained from AD patients [15], and the concentration of GS is significantly increased in AD CSF [16]. GS levels are also significantly higher in prefrontal cortex of AD patients than they are in non-demented controls [17]. Complicating the interpretation of these alterations in the amount of GS protein, the activity of GS is vulnerable to mixed-function oxidation which rises exponentially with age. Oxidized GS has reduced activity and is preferentially degraded [18]. This oxidation-induced loss of GS activity is brain region specific; it happens at double the rate in frontal lobe compared to the occipital lobe. Importantly, the decrease is more significant in frontal cortex from AD patients than from age-matched controls [19]. Proteomic analysis has identified GS as one of the cellular proteins most prone to oxidation after Aβ_1–42_ treatment *in vitro* [20]. *In vivo*, GS is one of the 3 hippocampal proteins (along with PIN1 and ENO1) that are found to be significantly oxidized in both MCI and AD compared to controls [13]. The suggestion is that although GS protein levels may rise, GS activity is compromised early in the Alzheimer's disease process, the deficit persists throughout the course of the disease, and perhaps like tau, the quantities in CSF increase as the GS protein in brain cells becomes increasingly dysfunctional.

In this study, we have explored the effect of glutamine and GS supplementation on the survival of both N2a cells and primary neurons. We compare the effects of altering endogenous glutamine (through manipulations of intracellular GS and its activity) with those found by altering the levels of exogenous glutamine. We document an unexpectedly complex relationship suggesting that both intracellular and extracellular glutamine are essential for neuronal health. To make the link to neurodegenerative disease, we extend these findings to the in *vivo* situation. We have used two mouse models of AD to test the effect of dietary supplementation of glutamine on the pathological features of the models. In the aggregate, our data suggest that glutamine may have significant neuroprotective effects that help restore homeostatic functions that are lost in AD.

## Materials and Methods

### Ethics Statement

Human frozen tissues and formalin-fixed sections were obtained from the Alzheimer's Disease Research Center, Washington University School of Medicine and the Alzheimer's Disease Research Center, Case Western Reserve University. Experimental procedures involving these samples were approved by the IRB of above two institutions and the Rutgers University IRB. All animal procedures carried out in this study were in accordance with Rutgers University IACUC standards, approval ID: 06-027.

### Human cases

Frozen brain tissues or formalin-fixed/paraffin-embedded 10 µm sections from AD and age-matched controls were obtained from the Alzheimer's Disease Research Centers at Case Western Reserve University and Washington University School of Medicine. Some of the cases used for this study had been clinically diagnosed with AD and were subsequently confirmed with standard pathological examination. Other cases died with no explicit diagnosis of their dementia, but were scored on neuropathological examinations as being either Braak stage V or VI. For the purposes of this study, we defined these cases as AD.

### Mouse strains

Two AD mouse models were examined [21] in this study. Both carry the entire human APP gene inserted into the mouse genome from a microinjected yeast artificial chromosome. Mice from the R1.40 line (B6•129-Tg(APPSw)40Btla/J) carry the human APP gene with the Swedish mutation for familial AD – K670N/M671L. Mice from the 8.9 line (B6•129S2-Tg(APP)8.9Btla/J) are similar to R1.40 animals but carry a wild type APP gene. Both transgenic lines express all mRNA and protein isoforms of the human gene in a correct spatiotemporal pattern.

### Primary neuron and N2a cell cultures

Embryonic cortical neurons were isolated by standard procedures. E16.5 embryonic cerebral cortices were treated with 0.25% Trypsin-EDTA and dissociated into single cells by gentle trituration. Cells were suspended in Neurobasal medium supplemented with B27 and 2 mM glutamine, then plated on coverslips or dishes coated with poly-L-Lysine (0.05 mg/mL). All cultures were grown for a minimum of 5 days *in vitro* (DIV) before any treatment. Murine neuroblastoma N2a cells were purchased from ATCC (Manassas, VA, USA) and cultured in standard DMEM media supplemented with FBS (2% for differentiation, 10% for routine passage) or Neurobasal+B27 with or without glutamine. Synthetic amyloid-β peptide (Aβ1–42) was purchased from AnaSpec, Inc. (San Jose, CA). Peroxide oxidized Aβ1–42 dimer was prepared from synthetic human Aβ1–42 (5 µM) by incubation in PBS in the presence of hydrogen peroxide plus Cu2+ as previously described [22].

### Glutamine synthetase expression constructs and transfection

An expression construct encoding human GS was purchased from Origene (Cat# SC118847, pCMV6-XL5-GLUL, GenBank accession No.: NM_002065). The disease-related mutation (1021C→T) was introduced by means of site-directed mutagenesis using the system developed by Mutagenex, Inc. The success of the mutagenesis was confirmed by DNA sequencing using a GS internal forward primer (Flk-F): 5′-GACCCCTTCCGTAAGGACCC and a vector reverse primer (pCMV6 Seq-R): 5′-TTAGGACAAGGCTGGTGGGCAC. Sub-confluent N2a cells were transfected with either Lipofectamine™ 2000 or Lipofectamine™ LTX with PLUS™ Reagent (Invitrogen) following the manufacturer's suggested procedure.

### Western blotting and antibodies

For Western blots, protein extracts from tissues or cultures were made in RIPA buffer with protease inhibitors and phosphatase inhibitors, and then separated in 4–20% SDS–PAGE gel.

All antibodies used are commercially available. Antibodies against GS, 53BP1, ATM, phospho-S1981-ATM (**^P^**S1981-ATM), cleaved caspase 6, LC3, LC3B,, PCNA, cyclin A, PSD-95 and VAMP2 were all purchased from Abcam. Antibodies against cleaved caspase 3, ATR, phospho-S428-ATR (**^P^**S428-ATR), and phospho-S807/811-retinoblastoma (**^P^**S807/811-RB) were from Cell Signaling. The Map2 antibody was purchased from Sigma. Antibodies against γH2AX, Tau3R, Tau4R and phospho-tau (AT8) antibodies are all mouse monoclonals purchased from Millipore or Thermo. Synaptophysin antibody was from Invitrogen. β-actin antibodies were from Santa Cruz Biotechnology. Iba1 antibody was from Wako (Japan). The specificity of these antibodies has been established previously and confirmed by us through the use of Western blots.

### Comet assay

The comet assay procedure was carried out using OxiSelect ™ Comet Assay Kit from Cell Biolabs following the procedure suggested by the manufacturer. Briefly, cultured cells were detached from the culturing vessels by trypsinization. Cells were then mounted in soft gels and electrophoresed for 15 minutes at 1 V/cm. After electrophoresis, slides were washed in water and dried at room temperature. Finally, slides were stained with Vista Green DNA dye (Cell Biolabs, Inc.) and visualized under a Leica DM5000B fluorescent microscope or a Zeiss LSM 510 confocal microscope using the FITC filter.

### Immunohistochemistry and immunofluorescence

Each mouse was transcardially perfused with 4% paraformaldehyde under deep Avertin anesthesia, after which the brain was dissected from the skull and post-fixed overnight. For most material, the brain was embedded in paraffin and sectioned at 10 µm on a rotary microtome. The human materials were formalin-fixed, paraffin-embedded and sectioned at 10 µm. These were treated as described previously [23]. For immunohistochemistry, all paraffin sections underwent antigen retrieval with high temperature citrate buffer for 20 min, and then soaked in 0.3% hydrogen peroxide to remove endogenous peroxidase activity. Primary antibody was diluted in 10% goat serum with 0.5% Tween-20. Primary antibodies were detected using biotinylated goat anti-rabbit or anti-mouse secondary antibody (1∶400), avidin-biotin complex horseradish peroxidase and peroxidase substrates including DAB, DAB+Ni and VIP (Vector Laboratories). Some sections were counterstained with hematoxylin QS. For immunofluorescence, mouse brains were embedded in OCT (Tissue-Tek) and sectioned on a cryostat at 10 µm. Sections were pretreated with citrate buffer for 10 minutes, and then incubated with primary antibody. Alexa linked secondary antibodies were used to detect the presence of the antigens. Stained sections were photographed and viewed at a final magnification of 200 using Leica Application Suite/Leica DM5000B.

### LDH assay and MTT assay

For both LDH assay and MTT assay, cells were cultured in media without phenol red. The LDH activity of the supernatant was measured by Promega CytoTox 96® Non-Radioactive Cytotoxicity Assay, according to the manufacturer's instructions, recording absorbance at 490 nm using absorbance at 650 nm as reference. Cell viability (MTT assay) was evaluated using a Promega CellTiter 96® Non-Radioactive Cell Proliferation Assay kit following the manufacturer's instructions, recording absorbance at 570 nm using absorbance at 650 nm as reference.

## Results

### Both endogenous and exogenous glutamine are required for neuronal cell survival

Neurons take up exogenous glutamine as part of the glutamate/glutamine cycle. Brain astrocytes clear glutamate from the synaptic cleft, convert it glutamine and secrete it to the extracellular space. Here it is picked up by neurons and converted back into glutamate for use as a transmitter or used as a precursor for other cellular components. Astrocytes typically maintain GS levels that are readily visualized by immunocytochemistry; in neurons under normal physiological conditions, GS is nearly undetectable. When exogenous glutamine levels drop, however, neurons can be induced to express GS [11]. Thus, although the levels of exogenous glutamine can be easily regulated, the levels of endogenous glutamine are a more complex function of uptake plus the activity of GS.

**Figure 1:**
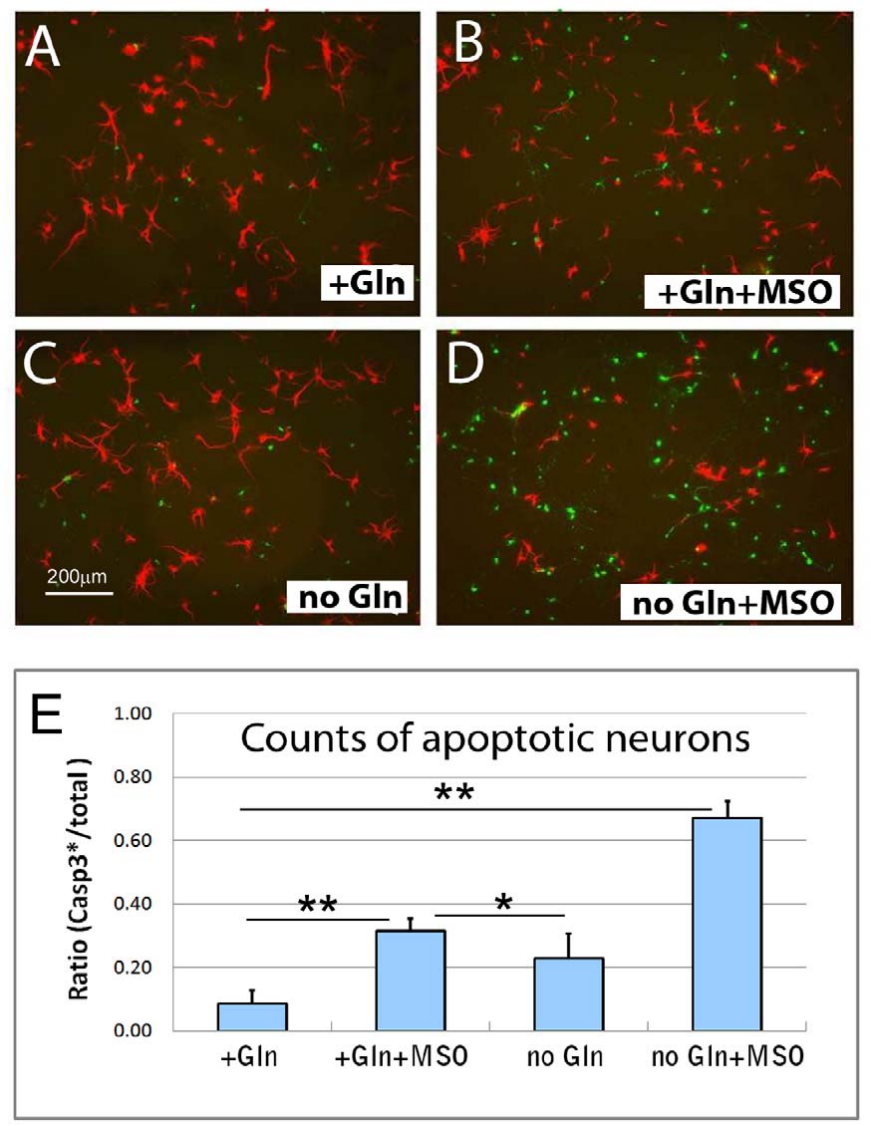
Deprivation of exogenous and endogenous glutamine synergistically promotes neuronal cell death. A–D) Primary neurons were grown in four different culture media. A) normal; B) normal glutamine in the presence of the glutamine synthetase (GS) inhibitor, methionine sulfoxide (MSO); C) glutamine-free; D) glutamine-free plus MSO. After 2–3 days cells were immunostained for Map2 (red) and cleaved caspase-3 (green), pictures were taken at a magnification of 200×. E) Cell counts reveal the ratio of apoptotic neurons (Caspase-3-stained) to total cell counts (DAPI staining). Error bars correspond to standard deviations; * denotes p<0.05, ** denotes p<0.01.

We found that in cultures of primary cortical neurons, reduction of exogenous glutamine had a small negative effect on neuronal viability (Figure 1A, C; quantified in Figure 1E). As the full effects of low media glutamine on the levels of intracellular glutamine might be offset by GS-induced glutamine synthesis, we tested the effects of the GS-inhibitor, methionine sulfoximine (MSO). MSO is the best known inhibitor of GS. It was originally isolated from nitrogenchloride-treated zein as the toxin responsible for the induction of convulsions, hysteria and epileptic fits in a number of animals (see reviews by Eisenberg et al. 2000) [24]. When added to neuronal culture media in the presence of 2 mM glutamine, MSO alone also caused the death of only a small number of neurons (Figure 1B, E). By contrast, when MSO was added to cells grown in glutamine-free medium (thus reducing both endogenous and exogenous glutamine), massive neuronal cell death was observed (Figure 1D, E). The reductions in exogenous and endogenous glutamine were synergistic. In normal medium there is a baseline level of caspase-3 labeling of about 7–8% of the total number of cells. MSO alone increased the rate of cell death by ∼3-fold; glutamine removal from the medium alone increased cell death by ∼2-fold. Applying MSO in the absence of glutamine, however, increased cell death by ∼8-fold. Thus, a healthy neuron requires glutamine both in the media and synthesized internally by the actions of GS.

### GS induction in hippocampal neurons from AD patients may be due to glutamine deprivation

**Figure 2:**
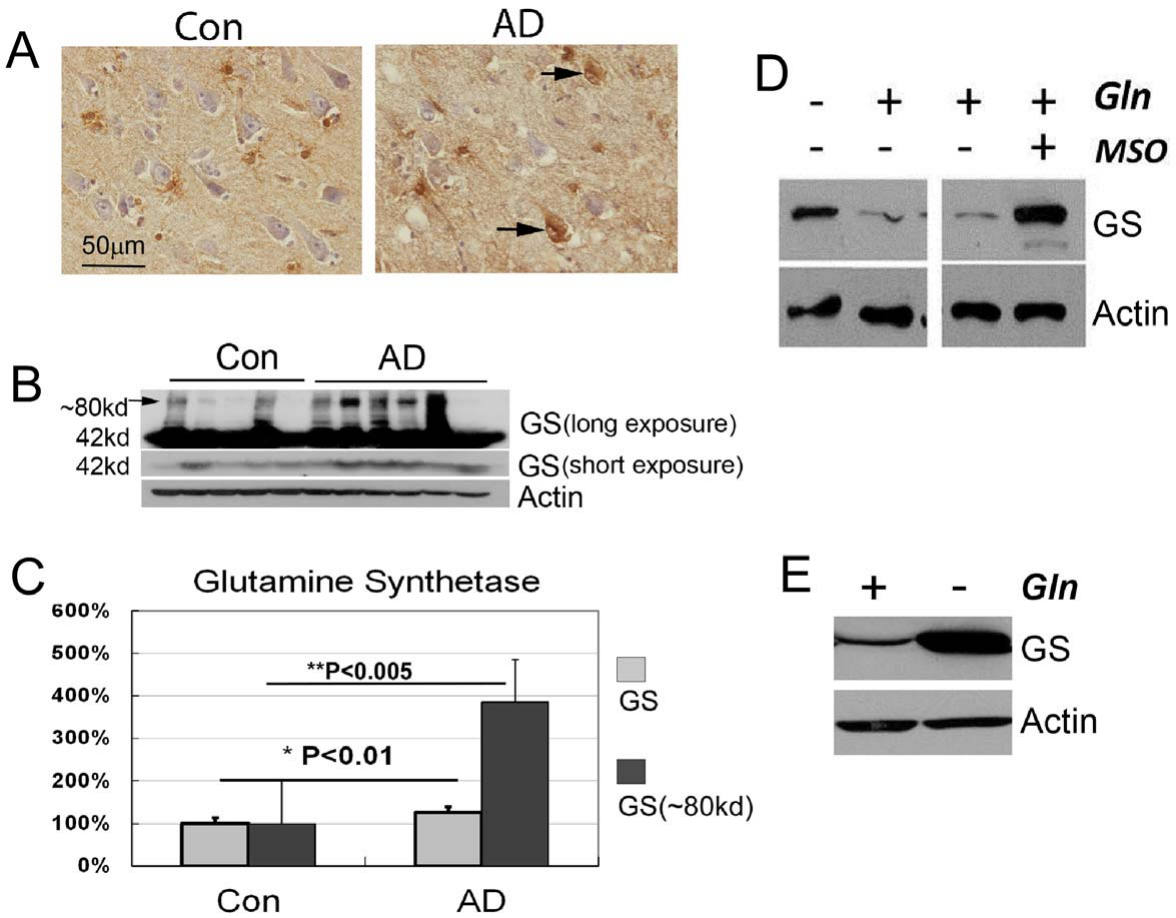
Glutamine deprivation up-regulates GS expression. A) GS immune-positive hippocampal neurons were detected in AD patients but not in control samples. Immunopositive astrocytes are found in both. B) GS levels in AD frontal cortex are significantly higher than in control samples. One representative blot at two different exposure times is shown. C) Quantification and statistics of 12 AD and 8 control cases. GS levels in controls were arbitrarily set as 100%. Error bars denote standard deviations. P values were calculated via Student's T-test. D) Removing glutamine from culture medium or inhibiting GS activity with MSO induces GS expression in primary neurons. E) Removing glutamine from the culture medium promotes GS expression in N2a cells.

In normal human hippocampus, astrocytes were the only cell type with detectable levels of GS by immunocytochemistry (Figure 2A). In AD patient samples, consistent with previous reports [25], we found numerous hippocampal neurons with elevated levels of GS (arrows, Figure 2A). Astrocyte staining was not noticeably altered in these AD samples. The percentage of GS-positive AD pyramidal neurons varied from subject to subject and from region to region, ranging from 1–50% of the total population. In the 4 control cases we examined, GS-positive neurons were rare (<1%). We validated these findings by Western blot using frozen brain samples from prefrontal cortex (Brodmann area 9 – BA9) of 20 additional subjects (12 AD; 8 control). As with the hippocampal immunohistochemistry, there was variability among the samples. These blots clearly indicate, however, that GS levels were elevated in frontal cortex of AD patients compared with controls (Figure 2B). When these results were averaged across all subjects, the increases in GS monomer and a higher molecular weight species that is likely to be a GS dimer were both highly significant (Figure 2C).

To verify that up-regulation of GS in neurons is triggered by glutamine deprivation, we cultured mouse primary cortical neurons (DIV 7–8) in Neurobasal media with or without glutamine, in the presence or absence of 5 mM MSO, for 3 days before collecting samples for Western blotting and immunocytochemistry. In normal Neurobasal medium with 2 mM glutamine, neurons express only trace amount of GS. As predicted by the *in vivo* work, GS expression increased robustly when primary neurons were deprived of glutamine either by removing exogenous glutamine from the media (Figure 2D, lane 1) or by blocking endogenous GS activity by MSO (Figure 2D, lane 4). The same relationships were found in N2a cells, a murine neuroblastoma cell line (Figure 2E).

### Cellular response to GS levels varies in different exogenous glutamine concentrations

**Figure 3:**
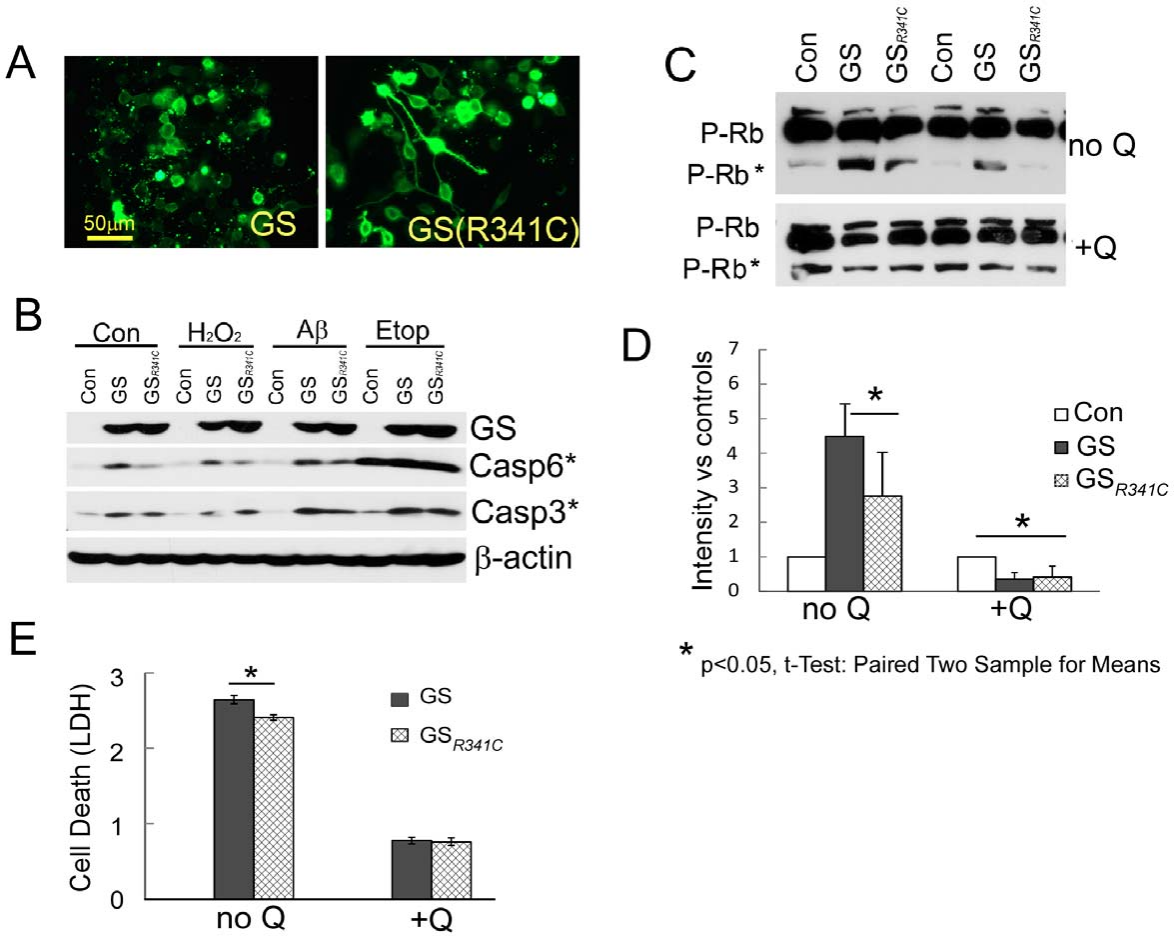
Overexpression of GS enhances neurodegeneration in the absence of exogenous glutamine. A) GS overexpression causes the degeneration of processes in differentiated N2a cells, while the low activity mutant GS^R341C^ appears to have no same effect. B) Both wild type and mutant GS overexpression increases the levels of apoptotic markers (cleavage of caspase-3 and caspase-6) in basal conditions and under stress tested including 0.1 mM H_2_O_2_, 100 nM Aβ and 20 µM etoposide. Mutant GS^R341C^ had consistently less effect than GS^WT^. C) Western blot shows the effects of GS overexpression in the presence or absence of free glutamine in the culture medium. The phospho-RB antibody provides evidence of both enhanced cell cycle (increased full length P-Rb) and cell death (caspase-3-cleaved Rb, P-Rb*) simultaneously. D) Quantification of 4 repetitions of the experiment illustrated in panel C. Error bars denotes standard deviations. * = p<0.05; ** = p<0.01 (by Student's T-test). E) Cell death evaluated by LDH assay. N2a cells overexpressing wild type GS and GS(R341C) were treated with 1 mM H_2_O_2_ for 30 minutes then allowed to recover in fresh media with or without glutamine for 6 hours before LDH assay. Error bars denote standard deviations. * = p<0.05.

Deprivation of exogenous glutamine can cause higher GS expression in neurons. The question we next addressed was whether this *de novo* expression of GS in neurons is protective or detrimental. Our results further underscore the complex nature of glutamine regulation in cells. We tested two GS constructs expressing the human GS transcript 1: wild type GS (GS^WT^) and an activity-deficient mutant, GS^R341C^. The GS^R341C^ construct harbors a point mutation, R341C (a T→C change at nucleotide 1021 of human GS transcript 1), and encodes a peptide with reduced GS activity [8]. N2a cells were transfected with either construct or treated with only transfection reagents as a negative control. After 24 hours, culture media were replaced with differentiation media (Neurobasal without glutamine). After another 24 hours, cells were treated with three different stressors – 100 µM H_2_O_2_, 100 nM oxidized Aβ_1–42_ (oxyAβ), or 5 µM etoposide – for 24 hours in the same media. Then cells were processed for Western blotting or immunocytochemistry. Extensive degeneration of both processes and cell bodies was observed in GS^WT^ but not GS^R341C^ -transfected cells (Figure 3A). The levels of cleaved caspase-3 and caspase-6 were substantially higher in cells with high GS activity (GS^WT^ transfection) than in cells expressing the GS^R341C^ mutant (Figure 3B), even though comparable amount of the two proteins were present. We repeated the transfections using cells grown in medium containing 2 mM glutamine. Intriguingly, with ample exogenous glutamine, high GS expression reduced caspase-3 activity – as measured by the cleavage of key cellular proteins such as retinoblastoma (RB, Figure 3C, D). To directly evaluate cell death we treated N2a cells with H_2_O_2_ and measured the lactate dehydrogenase (LDH) activity in culture media, The LDH results confirmed the caspase 3 and caspase 6 data (Figure 3E). First, low glutamine increases cell death and second, the difference between wild type and mutant GS is only seen in low glutamine. Viewed together, our data point to the conclusion that high levels of exogenous glutamine are highly beneficial to neuronal cells whereas treatments that lead to high GS activity may be useful only in certain conditions.

### Multiple stress responses are compromised without glutamine

**Figure 4:**
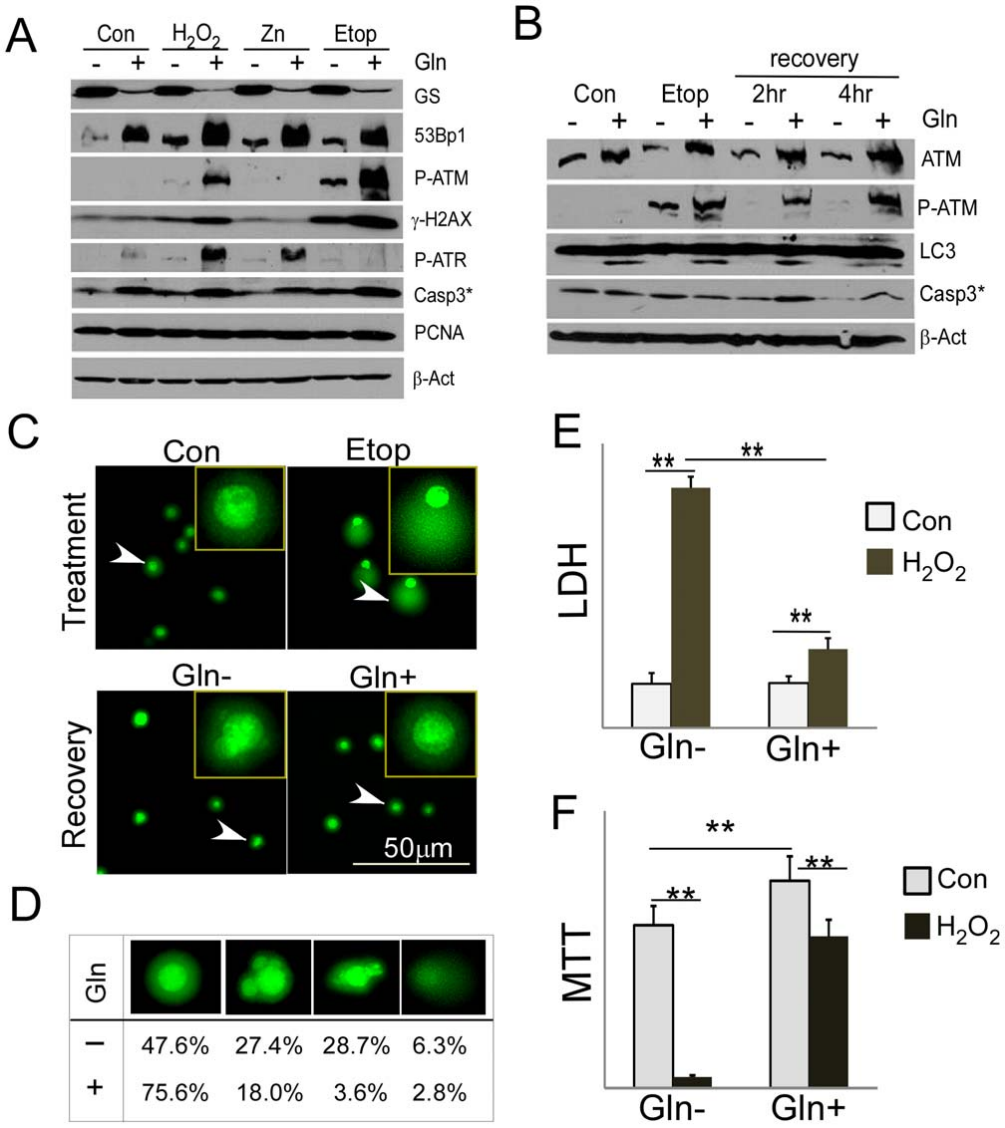
Stress responses are compromised without glutamine. A) N2a cells were treated with different cellular stressors for 30 minutes, and then collected and analyzed on Western blots. The various stress response proteins are indicated at the right and are described in more detail in the text. P-ATM = ^P^S1981-ATM. B) N2a cells were treated with 5 µM etoposide for 30 minutes to induce DNA damage. Etoposide was removed and fresh medium with or without glutamine was added. Cells were collected at indicated time points for Western blot analysis. Gln− = glutamine-free medium; Gln+ = 2 mM glutamine containing medium. C) Comet assays were performed on a portion of the samples collected for experiments shown in panel B. In each panel, the arrowhead points to a representative cell that is shown at higher magnification in the inset. There are few comet tails in control cultures. After 30 min in etoposide, however, the typical cell had well developed comet tails indicative of DNA damage. After 4 hours recovery, most comet tails disappeared in cultures with or without glutamine, but more pycnotic nuclei are found in glutamine deprived cultures. D) Counts of pycnotic nuclei as a measure of cell death. For each condition, 250 randomly selected cells were examined under a microscope; cells were separated into four categories: normal nucleus, blebbing nucleus, condensing nucleus, and disappearance of nuclear material. E) Cell death evaluated by LDH assay. N2a cells treated with 1 mM H_2_O_2_ for 30 minutes, and allowed to recover in fresh media for 6 hours. LDH activity was measured by the reactions absorbance at 490 nM, using absorbance at 650 nm as a reference. ** denotes p<0.01, error bars = standard deviation. F) Cell viability evaluated by MTT assay. Cells were treated as E, but allowed to recover for 24 hours before MTT assay (absorbance at 570 nm, reference at 650 nm). ** denotes p<0.01, error bars = standard deviation.

The results in Figure 3 show that cells in low glutamine are more sensitive to the effects of a variety of different stressors. To explore this effect further, we treated N2a cells grown in normal or reduced glutamine with H_2_O_2_ (1 mM – a model of oxidative stress); ZnSO_4_ (0.2 mM – a model of heavy metal toxicity); and etoposide (20 µM – a model of DNA damage). Cells were stressed for 30 minutes then harvested for Western blot analysis. The results in Figure 4A illustrate that cells grown in any medium can respond to each of these conditions. They up-regulate their levels of several stress response proteins such as 53BP1 and appropriately modify other proteins either by phosphorylation (ATM) or cleavage (caspase-3). The magnitude of all of these responses, however, is considerably diminished in low glutamine. For example, in all conditions, the levels of 53BP1 protein increased in response to stress. In low glutamine, however, the final levels of protein were significantly less than in normal glutamine. Activation of ATM and ATR by phosphorylation revealed a similar picture. Under oxidative stress or etoposide-induced DNA damage, the activating phosphorylation of both proteins increased. Etoposide treatment drove robust ATM auto-phosphorylation, this change in phosphorylation led to an actual change in ATM kinase activity can be seen in the increased levels of γH2AX, while the phosphorylation of ATR was driven preferentially by ZnSO_4_. For each of these measures of stress response, however, the magnitude of the response in low glutamine was significantly less than in normal exogenous glutamine. One likely reason for this difference is that the levels of total ATM and total 53BP1 are reduced in low glutamine (Figure 4A–B). Viewed as a whole, the data in Figure 4A suggest that in many different domains, cells in low glutamine are not well prepared for stress.

We presumed that the findings in Figure 4A reflected the reduced levels of the various damage response proteins. This assumes, however, that the levels of damage induced by the various stressors are equivalent in low glutamine. To address this concern, we examined the response of the cells to etoposide since DNA damage can be easily monitored by Comet assay. In the absence of etoposide, DNA damage is minimal, even in low glutamine (Figure 4C). After 30 min in 5 µM etoposide, DNA fragmentation is easily revealed by the appearance of significant comet tails (top row, Figure 4C). We then removed the etoposide and followed the cells' ability to repair their DNA. Within 4 hours, the Comet tails found on cells in both culture conditions disappeared, indicating the cells were able to join the majority of their DNA breaks, with or without glutamine (Figure 4C). Yet while the DNA might have been “repaired” in both situations, there was significantly more cell death in the glutamine-deprived cultures as assessed by the number of pycnotic nuclei (Figure 4D). The majority of this death is likely necrosis not apoptosis because markers of apoptosis (activated caspase-3) and autophagy (LC3-II) were higher in cultures with glutamine than in those without glutamine (Figure 4A, B). To directly evaluate cell death and viability, we also performed LDH assay after a 6 hour recovery (Figure 4E) and MTT assay after a 24 hour recovery (Figure 4F) from a 30 minutes H_2_O_2_ challenge. Collectively, glutamine is essential for the stress response and post damage survival of cells.

### Cells in low glutamine are more sensitive to the neurotoxic effects of Aβ

**Figure 5:**
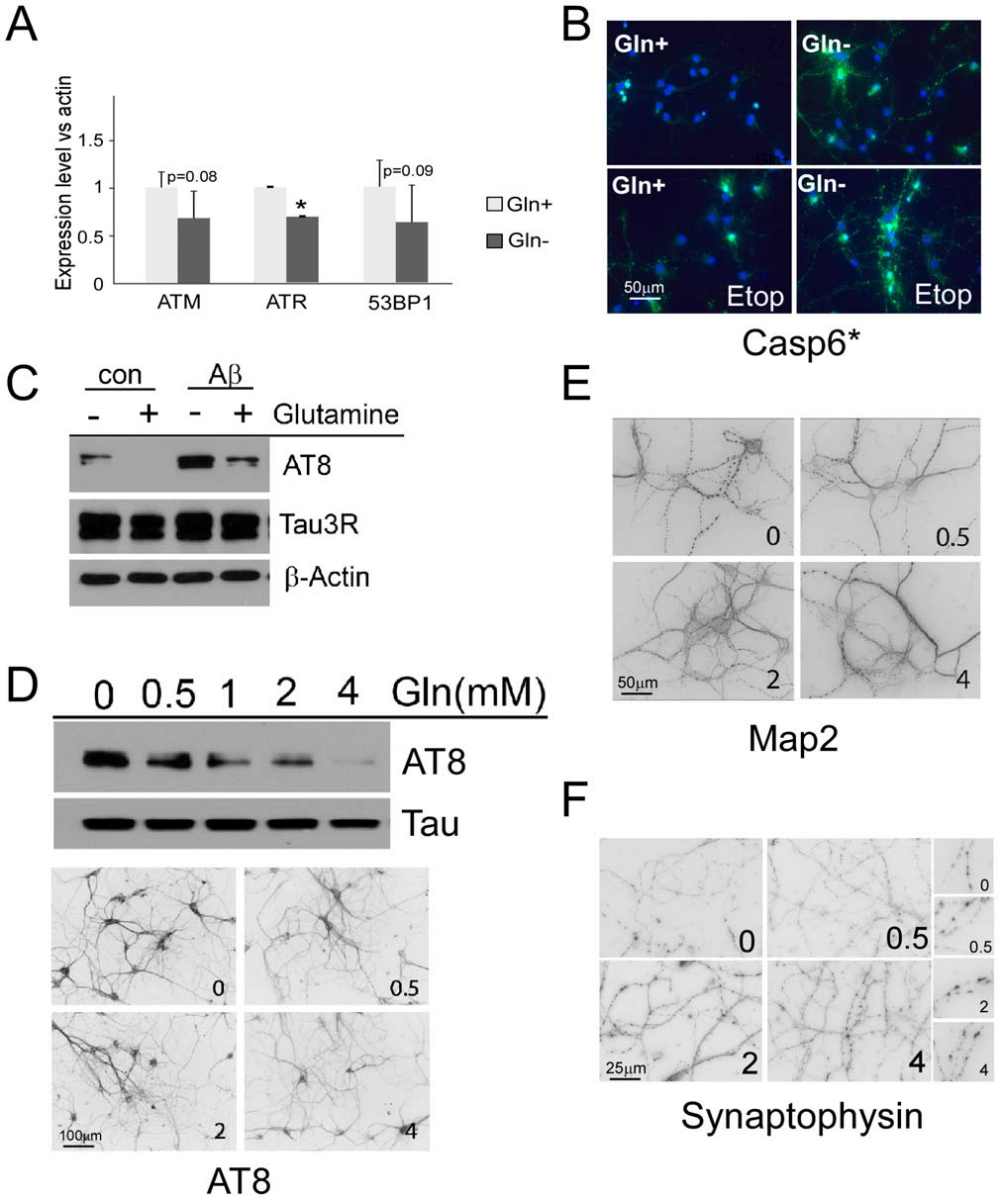
Neurons in low glutamine are more sensitive to the neurotoxic effect of Aβ. A) ATR levels are significantly reduced in neurons cultured without glutamine. ATM and 53BP1 are reduced by a similar amount, but the variability in the assay kept these change at the level of a trend rather than a significant difference. Average of 3 repeat experiments; error bar denotes standard deviation, * = p<0.05. B) Neurons grown for 9 days (DIV9) in Neurobasal medium with or without glutamine were treated with 20 µM etoposide for 30 minutes, then immunostained for activated caspase 6 staining (green) and counterstained with DAPI (blue). C) Aβ-induced tau phosphorylation in primary neurons depends on glutamine status in the culture medium. D) Tau phosphorylation is suppressed by glutamine in a concentration-dependent manner, even though levels of total tau (3R) remain unchanged. E) Aged neurons treated with Aβ have more processes degeneration (beaded Map2 stained dendrites) in the absence of glutamine. F) Synaptophysin staining is increased in the presence of glutamine. The numbers in each panel refer to the concentration of glutamine (mM) in the culture medium.

N2a cells grown in low glutamine are sensitive to toxic effect of DNA damage and oxidative stress, likely due to low expression of stress response proteins such as ATM and 53BP1(Figure 4A–B). However, this impaired response capability is not unique to the N2a cells; similar reductions of ATM, ATR and 53BP1 are detected in primary neurons deprived of glutamine (Figure 5A). Like N2a cells, neurons grown in low glutamine were more sensitive to DNA damage as well (Figure 5B). The importance of glutamine to the survival of neuronal cells prompted us to examine the effect of glutamine supplement on Aβ-induced toxicity. Primary neurons were cultured in medium with 2 mM glutamine (replaced every 2–3 days) until DIV 10–15. Cells were then re-fed with fresh medium with or without glutamine. Twenty-four hours later, the cells were treated with 100 nM oxyAβ for additional 24 hours; oxyAβ has been shown to more readily form neurotoxic oligomers and is more potent than un-oxidized Aβ in its ability to induce cellular stress in neurons [22]. We monitored the neuronal response using the AT8 phospho-tau antibody. Although oxyAβ induced tau phosphorylation in cultures with or without glutamine, the levels of phospho-tau were significantly reduced in the presence glutamine (Figure 5C). The effects of glutamine were dose dependent as can be seen from the effects on tau phosphorylation (Figure 5D). Even neurons in long-term established cultures responded to 3 days of glutamine deprivation and Aβ treatment by showing more processes beading (Figure 5E) and less synaptophysin staining (Figure 5F) suggesting reduced synaptic density.

### Glutamine supplementation appears to protect neurons *in vivo*

These *in vitro* findings provide multiple pieces of evidence that under conditions of neuronal stress such as is found in neurodegenerative disease, glutamine might have significant clinical value. The Aβ results further suggest that Alzheimer's disease might be a prime candidate for intervention. To test this concept in a preclinical disease model, we examined the effects of dietary glutamine on the phenotypes of the R1.40 and 8.9 transgenic mouse strains. Both lines carry an entire genomic copy of the region of human chromosome 21 with the APP gene [21]. The R1.40, mice develop amyloid plaques by 13 months (on a C57BL/6J background); 8.9 mice remain plaque-free throughout their lives on this background. Both lines develop neuronal cell cycle events in brain regions homologous to those affected in humans [26], [27]. These cell cycle events are robust outcome measures that are useful in tracking impending neurodegenerative events. They are induced by the AD-related brain inflammation as they can be prevented by NSAID treatment or advanced by an additional immune challenge such as LPS injection [28]. Given that LPS infusion in healthy human subjects causes a dramatic depletion of glutamine from their brains [29], we speculated glutamine may have a significant effect on LPS-treated R1.40 and 8.9 mice.

**Figure 6:**
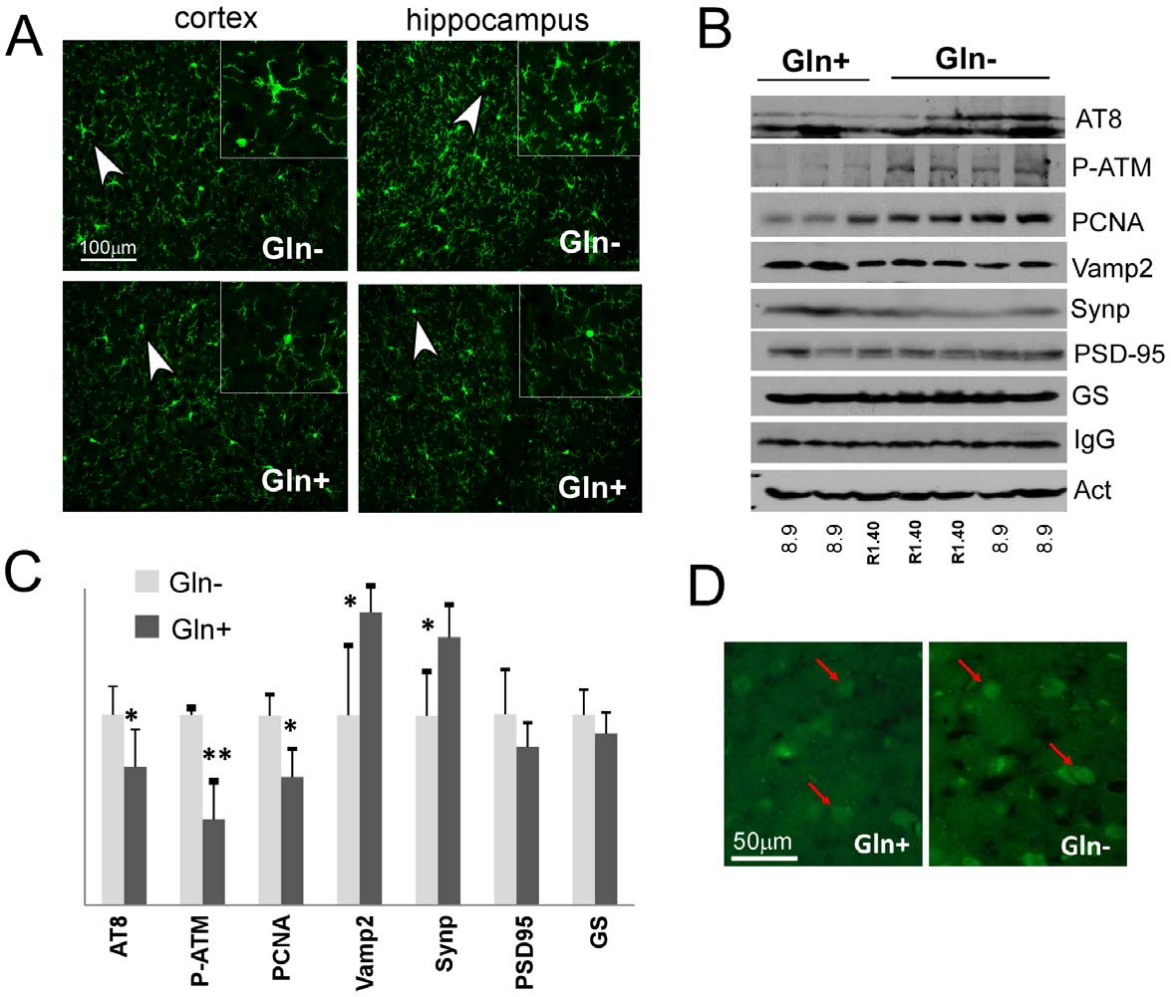
Glutamine supplementation protects neurons in AD mouse models *in vivo*. R1.40 and 8.9 mouse models of AD were treated with LPS as an immune challenge to enhance the degenerative phenotype. A) Microglia activation is dampened in mice supplemented with glutamine. Two different brain regions are shown as indicated at the top of each column. Microglia was stained with Iba-1 antibody. Activated microglia has thicker and shorter processes compared to resting microglia. B) Western blots show protective effect of glutamine supplementation (Gln+): lower tau phosphorylation (AT8), less cell cycle reentry (PCNA), less ATM activation (P-ATM) and more expression of synaptic maintenance proteins (VAMP2 and synaptophysin).C) Western blots shown in panel B were quantified and analyzed for statistical significance between control and glutamine supplementation, * denotes p<0.05. D) Immunofluorescent images show less cyclin A staining in cortical neurons of animals supplemented with glutamine.

One-year-old R1.40 and APP8.9 mice were fed 4% glutamine in their drinking water for 10 days. The following day they were injected with a low dose of LPS to induce systemic inflammation [28], and sacrificed 2 days later for analysis. Control mice were treated the same way except that they were kept on regular drinking water. We had 3 mice in the glutamine supplement group and 4 mice in the control group. We did extensive comparison of all the markers we used and found no differences between APP8.9 and R1.40. The only exception was the transgene encoded APP; R1.40 mice had higher APP expression than APP8.9 mice, as expected. Instead, we found a significant difference between mice with or without glutamine in their drinking water. As expected, in mice fed with normal drinking water (Gln−), LPS treatment was accompanied by microglial activation, as can be seen from the thickening and shortening of their Iba-1-stained processes (Figure 6A). In mice supplemented with glutamine, however, LPS failed to induce activation. Microglia in these mice (Gln+) showed the normal resting phenotype with thin and long Iba-1-positive processes. Glutamine supplementation also led to a reduced level of tau phosphorylation, as well as ATM phosphorylation (Figure 6B–C). The expression of the cell cycle maker, PCNA (Figure 6B–C) was also reduced with glutamine supplementation. Immunostaining using cyclin A antibody corroborated the PCNA results (Figure 6D, arrows). VAMP2, one of the synaptic vesicle SNARE proteins that is reduced both in AD models and in AD patients [29], was restored by glutamine supplementation, as was synaptophysin (Figure 6B–C). The levels of PSD95, however, remained unchanged. Thus, a 10-day regimen of glutamine supplementation was sufficient to block the expression of several indices of neurodegenerative disease in two AD mouse models.

## Discussion

Our data provide strong support for a neuroprotective effect of glutamine supplementation in a variety of different situations. The molecular nature of this protection is complex and reflects the levels intra- and extra-cellular glutamine as well as the levels and state of oxidation of the synthetic enzyme, glutamine synthetase (GS).

Neurons in normal situations express little GS, but when exogenous levels of glutamine fall, we find that neuronal GS expression increases significantly. This is consistent with repeated observations that the activity of GS is regulated by cumulative feedback inhibition and is dictated by the overall cellular demand for glutamine [30]. It is also consistent with the finding that patients with congenital glutamine deficiency resulting from GS mutations (R324C and R341C) have much higher expression of GS message than either their parents or normal controls [8]. As the specific activity of R324C and R341C is reduced from wild type, the suggestion is that GS activity, rather than protein concentration, is the attribute that regulates the level of GS transcription.

These observations have direct relevance for neurodegenerative disease as reduced glutamine/glutamate levels have been reported in the AD brain [31], [32], [33]. As might be predicted, in regions affected by AD pathology, many neurons are found to express abnormally high levels of GS (e.g., Figure 2A and earlier reports [25], [34]). Manipulating GS levels, however, carries risk as a therapeutic target. While, increasing GS might help to raise intracellular glutamine, in an environment of low exogenous glutamine degeneration is induced (Figure 3). Further, the complex chemistry of the AD brain worsens the situation since increased levels of catalytically active GS renders a cell hypersensitive to the toxic effects of Aβ and other stressors, an outcome to be avoided in AD.

Manipulation of glutamine itself would seem to have fewer such risks. In addition to the direct neuroprotective effects, there is a significant anti-inflammatory action of glutamine supplementation (Figure 6A). Perhaps because the immune system must overcome this effect to be fully reactive, the pro-inflammatory cytokine, TNFα, has been shown to reduce GS expression in astrocytes [10]. This would be consistent with observations that systemic inflammation induced by LPS injections in human volunteers reduces the glutamine pool in brain [35]. Chronic inflammation has emerged as a candidate for the one of the central pathogenic features of AD [36]. These data suggest, therefore that glutamine supplementation may be beneficial to AD patients as it should be anti-inflammatory in the brain. In addition, it would appear that glutamine supplementation improves the DNA damage response (Figure 4). This may well explain the success of glutamine supplementation in mitigating the harmful side effects of radiotherapy for various cancers [37], [38]. This effect also has relevance for late-onset diseases such as AD as it has been argued that DNA damage is a direct cause of aging [39]. The cellular DNA damage response system is a complex network of proteins that protect cells by either fixing the damage or forcing cells into apoptosis or senescence. If the repair process is blocked, however, some DNA will stay unrepaired and the cells will live. This phenomenon is more prevalent in non-replicating or slowly replicating cells, such as neurons in the brain, which cannot rely on DNA repair mechanisms associated with DNA replication. This is not a small problem. In humans, approximately 800 DNA lesions occur per hour in each cell – about 19,200 per cell per day [40]; and all pathways of DNA repair become less efficient with age [41]. ATM expression goes down dramatically in aging tissues [42] and is further reduced in AD frontal cortex compared to controls (unpublished data). Our observations that expression of both ATM and its downstream target, 53BP1, are substantially lowered in cells deprived of glutamine suggest that low glutamine supply may contribute to this phenomenon. Viewed in a therapeutic light, however, the same data provide reason to believe that glutamine supplementation may restore the aging brain to a more youthful state when measured by the capability of its stress responses.

Our data also show that glutamine deprivation reduces autophagy under both basal and stressed conditions (Figure 4B). This observation is consistent with a recent study showing that glutamine increases autophagy under basal and stressed conditions in intestinal epithelial cells [43]. In fact, autophagy and apoptosis are positive stress responses of cells that ensure the health of cells and organisms by replacing spent or damaged cellular components [44]. Given the fact that autophagy in AD is reduced, we can interpret these observations to mean that in this domain as well, glutamine supplement will be beneficial to the AD patients.

While our findings underline the complexity of an organism's response to fluctuations in the levels of glutamine, they also demonstrate that glutamine supplementation *in vitro* enables nerve cells to resist stresses similar to those that affect the human brain during the course of Alzheimer's disease. More intriguing still is our finding that glutamine supplementation may have a protective effect on AD pathogenesis *in vivo*. Two different mouse models of familial AD responded in positive ways to a relatively short (10-day) nutritional supplement of glutamine in their drinking water. The levels supplied were comparable to those used clinically in human subjects and the strong interference with the neurodegenerative phenotype of induced neuronal cell cycle events and lost synaptic markers suggests that this strategy might be a useful and cost-effective adjunct to other therapeutic measures. In summary, an optimal level of glutamine is important for aging neurons to respond to stress. Our data provide strong support for the protective effect of glutamine supplementation to avoid the neurodegeneration of Alzheimer's and other degenerative diseases.
